# Vertically ordered mesoporous silica film-assisted electrochemical cytosensor for the sensitive detection of HeLa cells

**DOI:** 10.3389/fchem.2023.1222067

**Published:** 2023-09-01

**Authors:** Zisan Zeng, Yang Zhao, Luoxing Yang, Fengna Xi, Danke Su

**Affiliations:** ^1^ Guangxi Medical University Cancer Hospital, Guangxi Medical University, Nanning, China; ^2^ Department of Chemistry, Key Laboratory of Surface and Interface Science of Polymer Materials of Zhejiang Province, Zhejiang Sci-Tech University, Hangzhou, China

**Keywords:** vertically ordered mesoporous silica film, cytosensor, HeLa cell, gated molecular transport, electrochemical detection

## Abstract

Designing fast and simple quantitative methods on cheap and disposable electrodes for the early detection of HeLa cells is highly desirable for clinical diagnostics and public health. In this work, we developed a label-free and sensitive electrochemical cytosensor for HeLa cell detection based on the gated molecular transport across vertically ordered mesoporous silica films (VMSFs) on the disposable indium tin oxide (ITO) electrode. As high affinity for a folate receptor existed on the membrane of HeLa cancer cells, folic acid (FA) functionalized VMSF could regulate the transport of electrochemical probe (Fe(CN)_6_^3−^) by the specific recognition and adhesion of HeLa cells toward the VMSF surface. In addition, VMSF, served as a solid skeleton, is able to effectively prevent the direct contact of cells with the underlying electrode, remaining the underlying electrode activity and favoring the diffusion of Fe(CN)_6_^3−^. Once specific adhesion of HeLa cells to the VMSF surface happens, Fe(CN)_6_^3−^ redox probe exhibits impeded transport in the silica nanochannels, ultimately resulting in the decreased electrochemical responses and realizing the quantitative determination of HeLa cells with a broad linear range (10^1^–10^5^ cells/mL) and a low limit of detection (4 cells/mL). The proposed electrochemical cytosensor shows a great potential application for the early diagnosis of cervical cancer.

## 1 Introduction

As one of the most common gynecological malignancy, cervical cancer has become the lethal cause of death among women in low- and middle-income countries (Feikin et al., 2006). Moreover, carcinoma cells can rapidly grow and spread to the adjacent parts or organs of the body, producing severe impact on women’s health. Clinical research indicates that the early detection and treatment of cervical cancer greatly contribute to improving cure rates and survival rates (Hou et al., 2022). At present, histopathological examination of biopsy specimens is the gold standard for the diagnosis of cervical cancer, which can acquire the information about the premalignant and malignant status of the cervix (Kundrod et al., 2019). However, biopsy specimens are obtained in an invasive way, which is inconvenient for clinical diagnoses (Li et al., 2020b). Morphological imaging technologies, including X-ray computed tomography and magnetic resonance technology, also play an important role for early detection and treatment of cervical cancer (Brindle, 2008). Nevertheless, these detection methods often depend on the expensive large-scale instruments and professional operators along with cumbersome operation steps (Wang et al., 2021). Therefore, it is particularly important to develop fast and sensitive detection methods for cervical cancer.

HeLa cells closely associated with cervical cancer are extremely important, and their content is helpful for the diagnosis and therapy of patients with cervical cancer (Gansukh et al., 2019). Traditional detection methods for HeLa cells include polymerase chain reaction (Lin et al., 2019), colorimetry (Chen et al., 2015; Teng et al., 2019), electrochemiluminescence (ECL) (Zhang et al., 2018), electrochemistry (Liu et al., 2021; Hu et al., 2022), photoelectrochemistry (Fan et al., 2019), and fluorometry (Guo et al., 2014). Electrochemical biosensors is equipped with prompt response, high sensitivity, and easy miniaturization, which has become one of the most powerful analytical techniques for complex biological samples (Gong et al., 2022a; Cui et al., 2023). In general, the specific recognition and electrochemical detection of cancer cells are accomplished by the utilization of tumor markers [folate receptor (FR), glycan receptors, and growth factor receptor] (Soleymani et al., 2020) or aptamer (Zhang et al., 2017). FR, as a kind of glycosyl-phosphatidylinositol-anchored cell-surface glycoprotein (O'Shannessy et al., 2012; Scaranti et al., 2020), will overexpress on the membrane of HeLa cells while limitedly express on that of normal cells, which has high affinity for folic acid (FA) (Scaranti et al., 2020). On the basis of the aforementioned case, FA has often been used as the recognition element to construct various electrochemical cytosensors for the detection of HeLa cells(Soleymani et al., 2018).

Vertically ordered mesoporous silica films (VMSFs) possessing ultrathin, ultrasmall and uniform pore size, perpendicularly aligned nanochannels, and high porosity are potential to provide high permeability and selectivity with respect to the size, charge, lipophilicity, and structure, which have attracted particular attention in the field of electrochemical analysis (Yan et al., 2020a; Ma et al., 2020; Yan et al., 2021; Han et al., 2022; Deng et al., 2023; Huang et al., 2023). With a high density of silanol groups on the silica walls, VMSF can either exhibit an electrostatic effect for the confinement of electrochemical/ECL probes (Gong et al., 2022b; Gong et al., 2022c; Zhou et al., 2023) inside the nanochannels or provide functional sites for the immobilization of specific recognition elements [e.g., antibody (Ma et al., 2022a; Ma et al., 2022b; Chen et al., 2022], aptamer (Wu et al., 2015; Zhang et al., 2023), and phenylboronic acid (Yan et al., 2020b) on the surface, which is able to construct various electrochemical/ECL sensors based on the surface-confined probes or the gated molecular transport of probes in solution across the silica nanochannels of VMSF (namely, “turn on” or “turn off” strategies) (Huang et al., 2022; Luo et al., 2022). In comparison with the immobilization of probes on the electrode surface, probes in solution combined with recognition element functionalized VMSF could realize all electrochemical reactions in a homogeneous solution without the complex electrode modification process. Moreover, compared to the antibody and aptamer, FA that served as the recognition element has a small size and long-term good stability, as well as high recognized ability for FR overexpressed on cancer cells (Xu et al., 2013). To the best of our knowledge, the utilization of FA functionalized VMSF for the electrochemical analysis of cancer cells has not been reported yet.

In this work, we report folic acid (FA) functionalized VMSF for highly sensitive and label-free determination of HeLa cells based on the gated transport of electrochemical redox (Fe(CN)_6_^3−^) across the nanochannels of VMSF. Cheap and disposable ITO electrodes are employed as the supporting electrode to stably grow VMSF. As high affinity for FR existed on the membrane of HeLa cancer cells, FA can be covalently immobilized on the VMSF surface by using EDC/NHS coupling agents. Once HeLa cells are specifically recognized and attached to the VMSF surface, the impeded transport of Fe(CN)_6_^3−^ in the silica nanochannels occurs, leading to the diminished electrochemical signals related to the concentration of HeLa cell. The quantitative analysis of HeLa cells can be realized by recording the DPV signals, and the fabricated electrochemical cytosensor with good biocompatibility shows a great potential application in the early diagnosis of cervical cancer.

## 2 Materials and methods

### 2.1 Chemicals and materials

Tetraethyl orthosilicate (TEOS), 3-aminopropyltriethoxysilane (APTES), hexadecyltrimethylammonium bromide (CTAB), potassium ferricyanide (K_3_ [Fe(CN)_6_]), potassium ferrocyanide (K_4_ [Fe(CN)_6_]), sodium phosphate dibasic dodecahydrate (Na_2_HPO_4_•12H_2_O), potassium hydrogen phthalate (KHP), bovine serum albumin (BSA), folic acid (FA), 1-ethyl-3-(3-dimethylaminopropyl)carbodiimide (EDC) N-hydroxysuccinimide (NHS), glucose (Glu), sodium chloride (NaCl), dopamine (DA), and ascorbic acid (AA) were all purchased from Aladdin Chemistry Co. Ltd., (China). Potassium chloride (KCl) and ethanol (EtOH) were ordered from Hangzhou Gaojingchem (China). L-alanine and sodium dihydrogen phosphate dehydrate (Na_2_H_2_PO_4_•2H_2_O) were obtained from Macklin (China). PBS (0.01 M) was prepared by mixing Na_2_HPO_4_•12H_2_O and NaH_2_PO_4_•2H_2_O into ultrapure water. Ultrapure water (18.2 MΩ cm) obtained from a Millipore water purification system was used to prepare all solutions. Therefore, all reagents used in the experiment were of analytical grade without further treatment. ITO-coated glasses were purchased from Zhuhai Kavio Optoelectronic Technology (China).

### 2.2 Apparatus and characterization

A transmission electron microscope (HT7700, Hitachi, Japan) was used to characterize the surface morphology of VMSF. VMSF was scraped moderately from the ITO electrode surface and subsequently dissolved in EtOH to obtain the transmission electron microscopy (TEM) specimen, which could be employed for the TEM observation operated at 100 kV. The Autolab electrochemical workstation (PGSTAT302N, Metrohm, Switzerland) was utilized to carry out all electrochemical experiments including cyclic voltammetry (CV) and differential pulse voltammetry (DPV). The scan rate for CV tests was 50 mV/s, and the parameters for DPV tests were as follows: step, 0.005 V; modulation amplitude, 0.025 V; modulation time, 0.05 s; and interval time, 0.2 s.

### 2.3 Modification of vertically ordered mesoporous silica films on the indium tin oxide electrode

The pretreatment of bare ITO was necessary to remove the impurity from the electrode surface and improve the hydrophilicity of the electrode surface for further VMSF growth. Briefly, bare ITO electrodes (5 cm × 0.5 cm) were soaked into NaOH (1 M) overnight and treated successively with acetone, ethanol, and deionized water under ultrasonication for 30 min. VMSF with good biocompatibility was grown onto the cleaned bare ITO electrodes by using electrochemically assisted self-assembly (EASA) approach as previously reported (Zhang et al., 2022; Zhou et al., 2022; Zou et al., 2022). The directly obtained electrode remains templated surfactant micelles (SM), termed as SM@VMSF/ITO. To remove SM from the silica nanochannels, HCl-EtOH (0.1 M) was employed and VMSF/ITO electrode was acquired.

### 2.4 Preparation of folic acid-aminopropyltriethoxysilane/vertically ordered mesoporous silica films/indium tin oxide electrode

FA with high affinity for FR existed on the membrane of HeLa cancer cells was functionalized on the surface of VMSF according to the previous literature with a slight adjustment (Li et al., 2020a), which is involved in the synthesis of FA functionalized with APTES (FA-APTES) and its covalent modification to the VMSF. First, FA (40 mg), EDC (20 mg), and NHS (30 mg) were dissolved into DMSO (2 mL) and stirred in the dark for 30 min to activate the carboxyl groups of FA and form NHS ester. APTES (125 μL) was then added to the aforementioned activated FA solution under stirring for 2 h to replace the active NHS esters, eventually resulting in the formation of the FA-APTES complex. Subsequently, the VMSF/ITO electrode was placed into EtOH (10 mL) containing FA-APTES (400 μL) and reacted for another 2 h. After being rinsed thoroughly with EtOH to remove unreacted FA-APTES, FA-APTES-modified VMSF on the ITO electrode was obtained called FA-APTES/VMSF/ITO.

### 2.5 Cell culture

HeLa cells were cultured in Dulbecco’s Modified Eagle Medium (DMEM) supplemented with fetal bovine serum (FBS, 10%) at 37°C in a humidified atmosphere containing CO_2_ (5%). The cells were trypsinized and subcultured every 2 days. The cell number was detected using a Petroff-Hausser cell counter.

### 2.6 Electrochemical determination of HeLa cells

The cells were separated from the medium by centrifugation at 2,000 rpm for 3 min and then washed twice with sterile PBS. The sediment was carefully redispersed in PBS to obtain a homogeneous HeLa cell suspension at a certain concentration. Then, the FA-APTES/VMSF/ITO electrode was incubated with various concentrations of HeLa cell suspension for 20 min. The steric hindrance effect produced in the recognition process inhibits the mass transfer of the probe through bulk solution to the underlying ITO electrode, leading to the reduction of the electrochemical signal and further realizing the detection of HeLa.

## 3 Results and discussion

### 3.1 The construction of the electrochemical cytosensor

**FIGURE 1:**
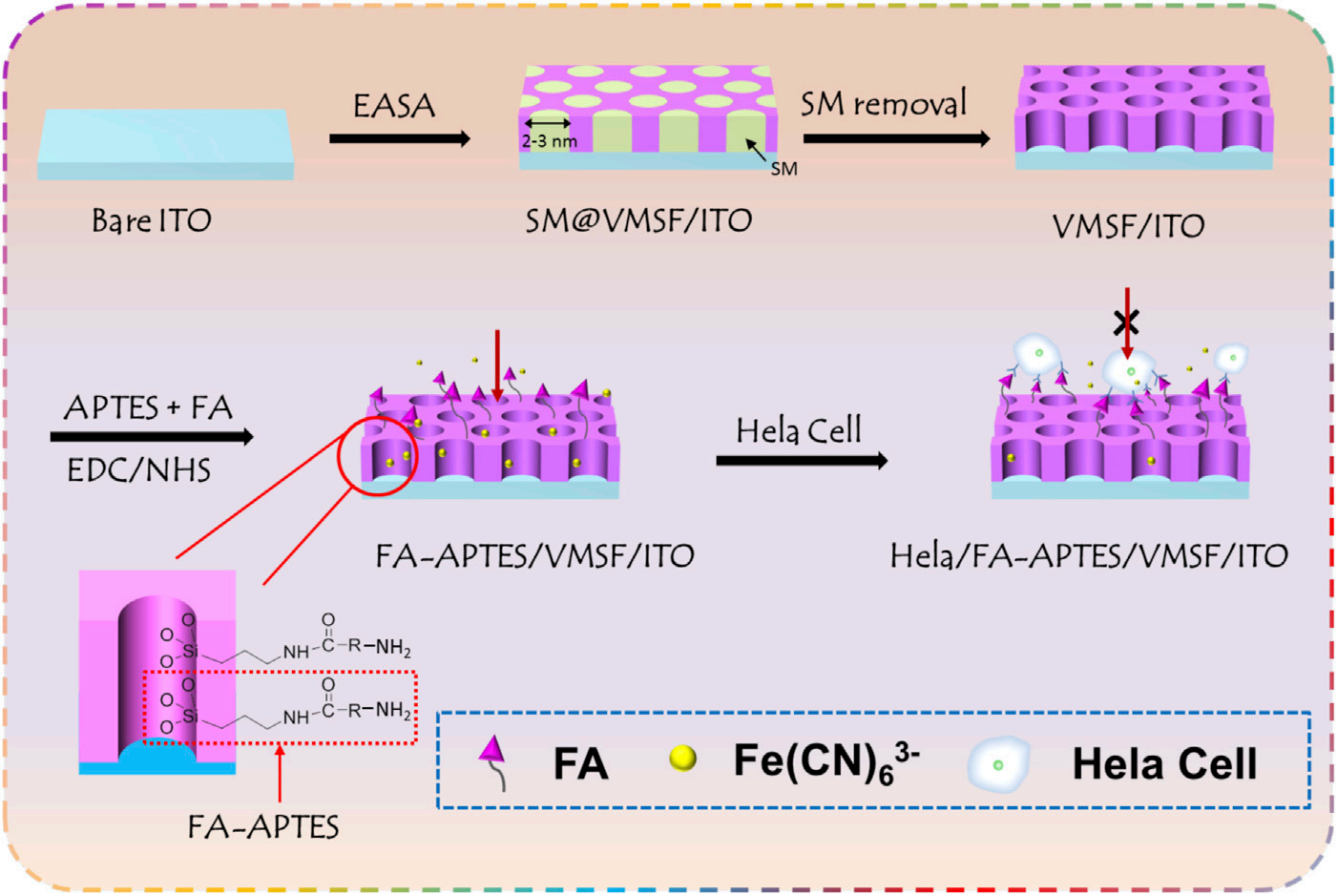
Schematic illustration for the fabrication of VMSF-assisted electrochemical cytosensor for HeLa cell detection.

Figure 1 shows the fabrication of VMSF-assisted electrochemical cytosensor for HeLa cell detection. As shown, ITO modified with VMSF (VMSF/ITO) was first obtained by using the EASA method and subsequent removal of SM, which could provide a gated-controlled electrode substrate for FA-APTES cross-linking and further HeLa cell recognition. Due to the amino groups of APTES and carboxyl groups of FA, FA could be functionalized with APTES through EDC/NHS activated agents, followed by modification to the surface of VMSF/ITO through the silanization reaction. Thus, FA possessing high affinity to FR-riched HeLa cells has been successfully grafted to the sensing interface, and the obtained sensor was termed as FA-APTES/VMSF/ITO. Benefiting from the overexpression of FR on the membrane of HeLa cells, HeLa cell could be specifically recognized by the proposed FA-APTES/VMSF/ITO sensor and attached to the electrode surface, producing a steric hindrance effect and eventually inhibiting the access of the electrochemical redox probe (Fe(CN)_6_^3-^) to the underlying ITO electrode. Therefore, the reduced electrochemical signals have a relation to the concentration of HeLa cells, allowing the quantitative determination of Hela cells. It should be noted that VMSF acting as a solid skeleton is capable of preventing the direct contact of cells with the underlying electrode, effectively maintaining the electrode activity and effective diffusion of the probe.

### 3.2 Morphology and electrochemical characterization of vertically ordered mesoporous silica films

**FIGURE 2:**
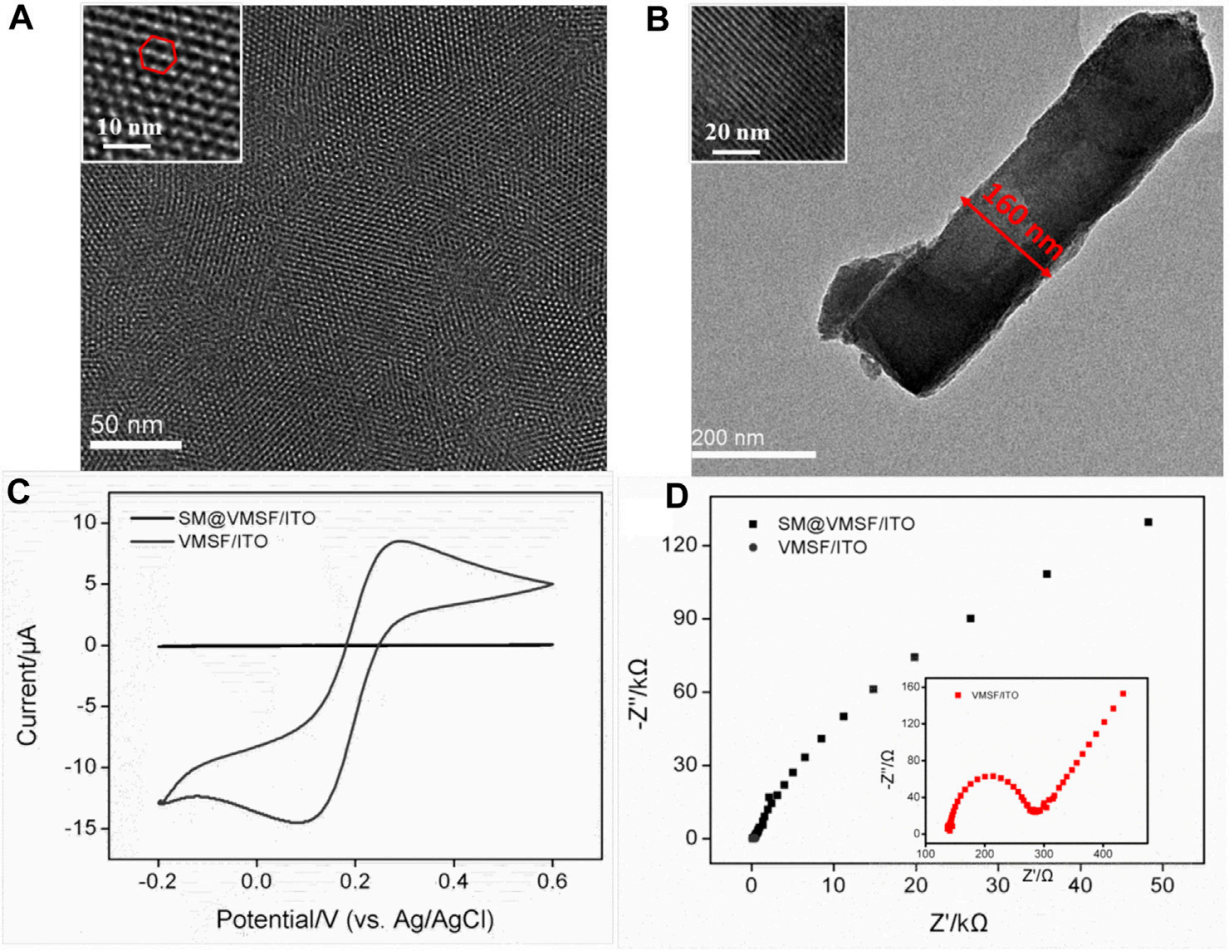
**(A)** Top-view and **(B)** cross-sectional TEM images of VMSF. Insets in **(A)** and **(B)** display the corresponding amplified TEM images. **(C)** CV curves obtained at the SM@VMSF/ITO and VMSF/ITO electrodes in KHP (0.05 M) solution containing Fe(CN)_6_^3−^ (0.5 mM). **(D)** Nyquist plots of SM@VMSF/ITO and VMSF/ITO electrodes obtained in a KCl (0.1 M) solution containing K_3_Fe(CN)_6_/K_4_Fe(CN)_6_ (2.5 mM).

The pore size and thickness of VMSF could first be confirmed from the top-view and cross-sectional TEM images shown in Figures 2A,B. It can be seen that the prepared VMSF with hexagonally oriented pores is intact without large defects, and the parallel nanochannels are in long-range order. The diameter and thickness are approximately 2–3 nm and 160 nm, respectively. Electrochemical strategies including CV and EIS were used to survey the integrity and permeability of VMSF. Figures 2C,D show that the SM@/VMSF/ITO electrode only displays charging currents and a large charge transfer resistance (*R*ct), which is attributed to the hydrophobic environment inside the nanochannels of VMSF formed by templated SM and further implies that the as-prepared VMSF is unbroken. Upon SM removal, the entrance of Fe(CN)_6_^3−^ through the silica nanochannels is unhindered and its redox reaction occurs at the underlying ITO electrode surface, exhibiting a pair of reversible redox peaks in the CV curve and a minor *R*_ct_ in the Nyquist plot. This is because the open nanochannels of VMSF assembled on the ITO electrode are beneficial for charge transfer. Previous results indicate the successful preparation of VMSF attached to the ITO electrode surface, giving rise to the suitable electrode interface for the immobilization of specific recognition element and cell adhesion.

### 3.3 Construction of the proposed electrochemical cytosensor

**FIGURE 3:**
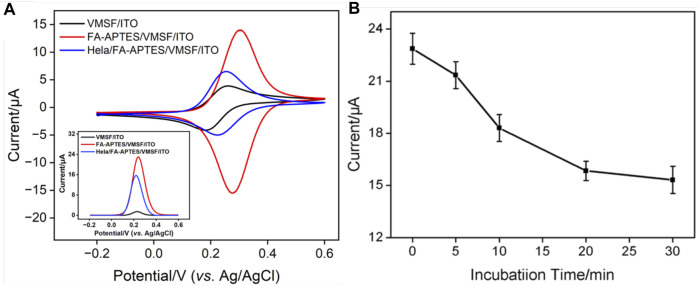
**(A)** CV curves of VMSF/ITO, FA-APTES/VMSF/ITO, and HeLa/FA-APTES/VMSF/ITO electrodes in KCl (0.1 M) solution containing Fe(CN)_6_^3−^ (0.05 mM). Inset is the corresponding DPV curves. **(B)** Optimization of the incubation time for HeLa cells on the proposed FA-APTES/VMSF/ITO cytosensor. Concentrations of Fe(CN)_6_^3−^ or HeLa cells are 0.05 mM and 10^4^ cells/mL.

The cytosensor interface was constructed using FA as a recognition element. Figure 3A shows the CV curves of VMSF/ITO, FA-APTES/VMSF/ITO and HeLa/FA-APTES/VMSF/ITO electrodes in KCl (0.1 M) solution containing Fe(CN)_6_^3−^ (0.05 mM). It could be found that a pair of reversible redox peaks corresponding to the electrochemical reaction of Fe(CN)_6_^3−^ is displayed at the VMSF/ITO electrode. After the modification of FA-APTES composite on the VMSF surface, the redox peak currents are significantly increased, which is attributed to the electrostatic effect by protonated amino groups on the FA-APTES composite. Once HeLa cells are identified by the FA-APTES/VMSF/ITO cytosensor, the redox peak currents remarkably decrease, which is due to the steric hindrance effect generated by cells attached to the electrode surface and the inhibited mass transfer of Fe(CN)_6_^3−^. The corresponding DPV curves are shown in the inset of Figure 3A, proving the successful construction of our FA-APTES/VMSF/ITO cytosensor.

### 3.4 Condition optimization of the proposed electrochemical cytosensor

As experimental condition has a crucial impact on the electrochemical performance of the fabricated FA-APTES/VMSF/ITO cytosensor, the incubation time for HeLa cells is optimized. Figure 3B shows the electrochemical responses at the FA-APTES/VMSF/ITO electrode under various incubation time periods for HeLa cells. It could be found that the anodic peak current of Fe(CN)_6_^3−^ depletes with an increase in the incubation time, and no evident variation is observed after 20 min, suggesting that HeLa cells have fully reacted with the binding site of the FA-APTES/VMSF/ITO electrode. Therefore, 20 min is considered as the appropriate incubation time in the following study. Moreover, the kinetic of the Fe(CN)_6_^3−^ reaction at the VMSF/ITO electrode was studied, and the results were shown in Supplementary Figure S1. As seen, the equilibrium time is 20 min, which is enough for the binding of HeLa cells on the FA-APTES/VMSF/ITO electrode.

### 3.5 Determination of HeLa cells

**FIGURE 4:**
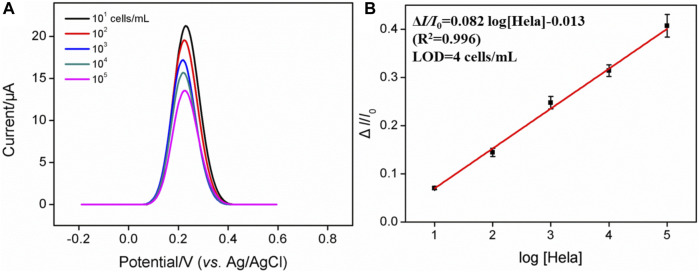
**(A)** DPV curves of the FA-APTES/VMSF/ITO cytosensor in 0.05 mM K_3_ [Fe(CN)_6_] and containing different concentrations of HeLa cells. Concentration range is 10^1^–10^5^ cells/mL. **(B)** Corresponding calibration curves for the detection of HeLa cells.

Figure 4 shows that when 0.05 mM Fe(CN)_6_^3−^ is present, the dynamic concentration range is from 10^1^ to 10^5^ cells/mL, and the obtained linear regression equation is Δ*I*/*I*_0_ = 0.082 log [HeLa] (cells/mL)−0.013 (*R* ^2^ = 0.996) with a limit of detection (LOD) of four cells/mL. (Δ*I* and *I*_0_ are defined as Δ*I* = *I*_FA_−*I*_Hela_ and *I*_FA_, respectively; *I*_FA_ and *I*_Hela_ represent the current values of the developed cytosensor before and after the incubation with various concentrations of HeLa cells). As displayed in Supplementary Figure S2, when the concentration of Fe(CN)_6_^3−^ is 0.5 mM, the dynamic concentration range is from 10^2^ to 10^6^ cells/mL, and the obtained linear regression equation is Δ*I/*Δ*I*_0_ = 0.17 log [HeLa] (cells/mL)−0.0042 (*R* ^2^ = 0.991) with an LOD of 12 cells/mL. On the contrary, a high concentration of Fe(CN)_6_^3−^ is able to produce the linear range at the high concentration range, while the low concentration of Fe(CN)_6_^3−^ generates high sensitivity and low LOD, which is more suitable for practical applications. Table 1 shows the comparison between previously reported electrochemical cytosensors with our developed FA-APTES/VMSF/ITO cytosensor. By contrast, our proposed electrochemical cytosensor is timesaving and has good detection performances in terms of a wide linear range and a low LOD.

**TABLE 1:** Comparison with the existing electroanalytical techniques for the detection of HeLa cells.

Strategy	Technique	Incubation time (min)	Linear range (cells/mL)	LOD (cells/mL)	Refs.
FA/Pt@BSA^1^/AuE	DPV	120	28−2.8 ×10^6^	9	Hu et al. (2022)
FA/MUA^2^/Au/GE^3^	EIS	/	6−10^5^	6	Wang et al. (2012)
FA/PAMAM^4^/Glu^5^/Cys^6^/AuNP/GCPE^7^	CV	60	10^2^−10^6^	100	Tepeli et al. (2015)
FA@UiO-66^8^/GE	EIS	60	10^2^−10^6^	90	Du et al. (2019)
FA films/PAH^9^/ITO	EIS	60	50−10^6^	4	Correia et al. (2021)
	CV		10^2^−10^5^	19	
FA/MUA/Au/BDD^10^	EIS	20	10^1^−10^5^	10	Weng et al. (2011)
FA-APTES/VMSF/ITO	DPV	20	10^1^−10^5^	4	This work

^1^Bovine serum albumin.

^2^11-Mercaptoundecanoic acid.

^3^Gold electrode.

^4^C12 dendrimer generation 4 solution.

^5^Glutaraldehyde.

^6^Cysteamine.

^7^Glassy carbon paste electrode.

^8^Zirconium MOFs.

^9^Poly(allylamine) hydrochloride.

^10^Boron-doped diamond.

### 3.6 Selectivity of the electrochemical cytosensor

**FIGURE 5:**
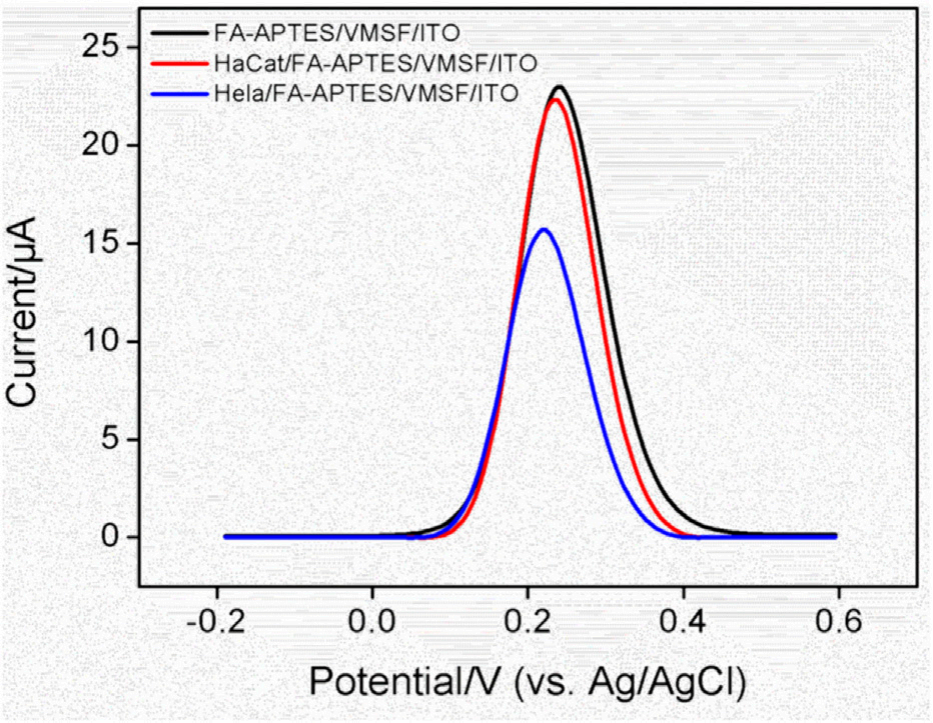
DPV curves of the FA-APTES/VMSF/ITO cytosensor in the absence and presence of HaCat (10^4^ cells/mL) or HeLa cells (10^4^ cells/mL).

The effect of potentially co-existed interfering substances including glucose (Glu), Na^+^, K^+^, Cl^−^, dopamine (DA), ascorbic acid (AA), alanine, and BSA, on the detection of HeLa cells was first evaluated using the FA-APTES/VMSF/ITO cytosensor. As shown in Supplementary Figure S3, only HeLa cells can produce an apparent signal at the FA-APTES/VMSF/ITO cytosensor. Then, selectivity of the fabricated FA-APTES/VMSF/ITO cytosensor was evaluated by comparing the DPV response of the normal cell (HaCat cell). Figure 5 shows the DPV curves of the FA-APTES/VMSF/ITO cytosensor toward the same concentration of HaCat cell and HeLa cell. As displayed, no evident anodic peak current variation is observed for the HaCat cell, while an evident decreased current for the HeLa cell is observed, demonstrating the excellent selectivity of the as-prepared FA-APTES/VMSF/ITO cytosensor.

**FIGURE 6:**
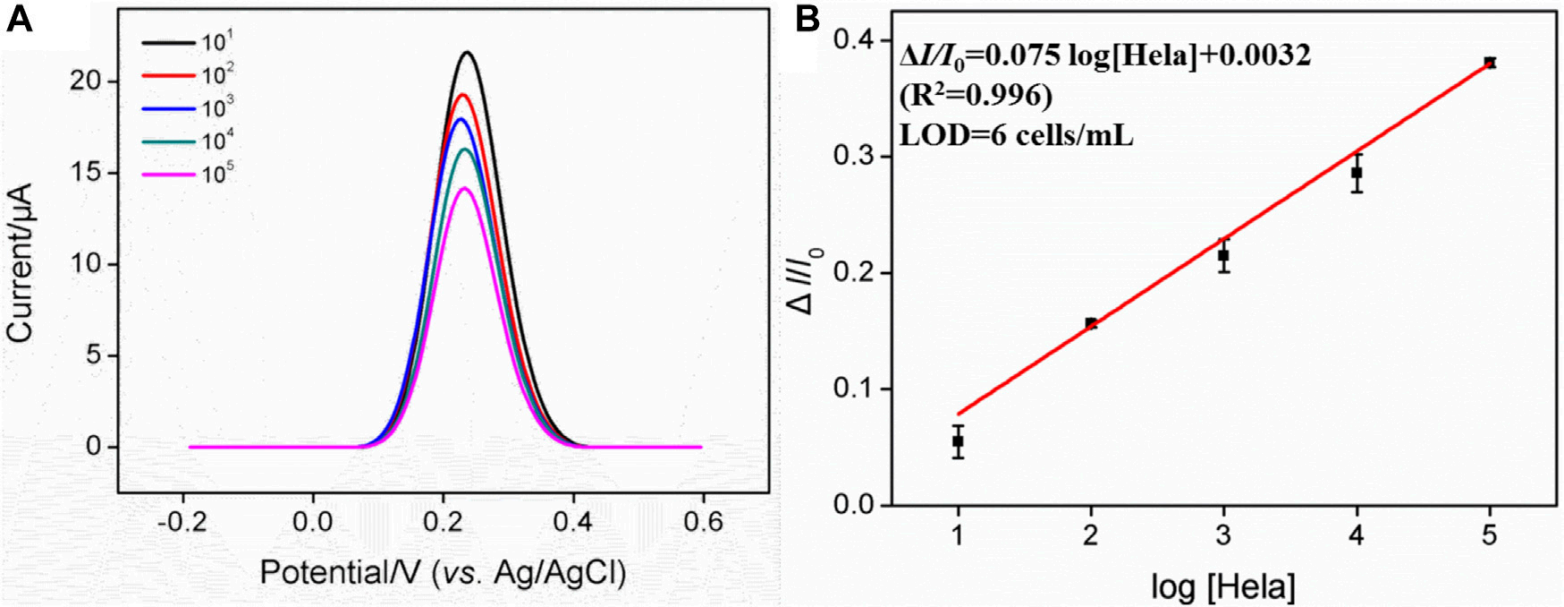
**(A)** DPV curves of the FA-APTES/VMSF/ITO cytosensor in 0.05 mM K_3_ [Fe(CN)_6_] containing 10^3^ cells/mL HaCat cells and different concentrations (10^1^–10^5^ cells/mL) of HeLa cells. **(B)** Calibration curve for the detection of HeLa cells.

To further examine the ability of the FA-APTES/VMSF/ITO cytosensor to distinguish between cancer cells and normal cells, HeLa cells with different concentrations were determined in the presence of HaCat cells, and the results were shown in Figure 6. As presented, in the range from 10 to 105 cells/mL, the anodic peak current gradually decreases with the increasing concentration of HeLa cells, and the fitting linear regression equation is Δ*I*/*I*_0_ = 0.075 log [HeLa] (cells/mL) + 0.0032 (*R* ^2^ = 0.996). Hence, the LOD is calculated as six cells per mL. The sensitivity for the detection of HeLa cells in the presence of HaCat cells is very close to that in buffer solution, further indicating that the FA-APTES/VMSF/ITO cytosensor fabricated in this work has high selectivity. However, our proposed cytosensor could not distinguish a specific kind of cell.

### 3.7 Reproducibility and stability of the electrochemical cytosensor

**FIGURE 7:**
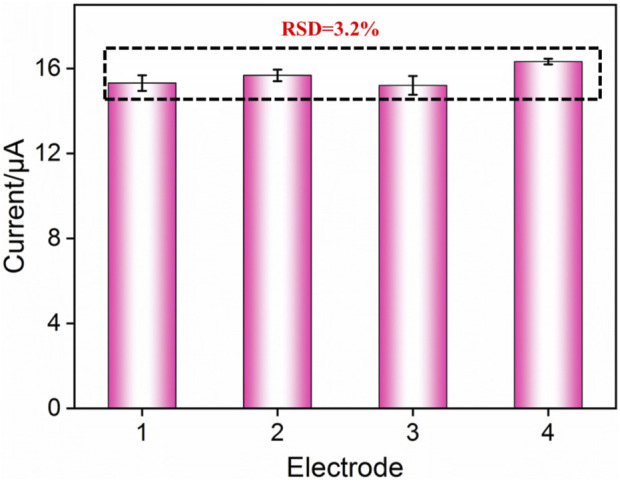
Anodic peak currents obtained from four different electrodes.

The reproducibility of the FA-APTES/VMSF/ITO electrochemical cytosensor was assessed by testing four sensors prepared in parallel. Figure 7 shows the relative standard deviation (RSD) of current values obtained from these four cytosensors in the presence of 10^4^ cells/mL HeLa cells is 3.8%, confirming that the as-prepared FA-APTES/VMSF/ITO cytosensor has satisfactory reproducibility. Moreover, after a 20-day storage, the developed FA-APTES/VMSF/ITO cytosensor still shows a stable signal for the detection of 10^4^ cells/mL HeLa cells.

## 4 Conclusion

In summary, a label-free and sensitive electrochemical cytosensor for HeLa cells has been constructed by grafting FA to the VMSF surface. FA has high affinity for FR on the membrane of HeLa cells and is able to confer VMSF with specific recognition capacity. When HeLa cells are specifically attached to the VMSF surface, the diffusion of Fe(CN)_6_^3−^ through the silica nanochannel is excluded, leading to the decreased electrochemical response and further allowing the quantitative analysis of HeLa cells with a wide concentration range and a low LOD. Furthermore, the fabricated electrochemical cytosensor has good selectivity, reproducibility, and biocompatibility, which presents a great potential application for the early diagnosis of cervical cancer.

## Data Availability

The raw data supporting the conclusion of this article will be made available by the authors, without undue reservation.
