# Supplementary material for: Vertically ordered mesoporous silica film-assisted electrochemical cytosensor for the sensitive detection of HeLa cells
Source: Front Chem. 2023 Sep 1;11:1222067. doi: 10.3389/fchem.2023.1222067 (PMC10506308; doi:10.3389/fchem.2023.1222067)
Supplement: Supplementary file 1 [file DataSheet1.docx]

**Vertically-ordered mesoporous silica films-assisted electrochemical cytosensor for sensitive detection of Hela cells**

**Zisan Zeng^1^, Yang Zhao^1^, Luoxing Yang^2^, Fengna Xi^2,*,^ and Danke Su^1,*^**

^1^ Guangxi Medical University Cancer Hospital, Guangxi Medical University, Nanning, China

^2^ Department of Chemistry, Key Laboratory of Surface & Interface Science of Polymer Materials of Zhejiang Province, Zhejiang Sci-Tech University, Hangzhou, China.

*** Correspondence:**

Corresponding authors: Fengna Xi and Danke Su

**Table of Contents**

**S1. Kinetic of the VMSF/ITO electrode reaction**

**S2. Optimization of Fe(CN)_6_^3−^ concentration**

**S3. Selectivity of the FA-APTES/VMSF/ITO electrode**

**S1. Kinetic of the VMSF/ITO electrode reaction**

**
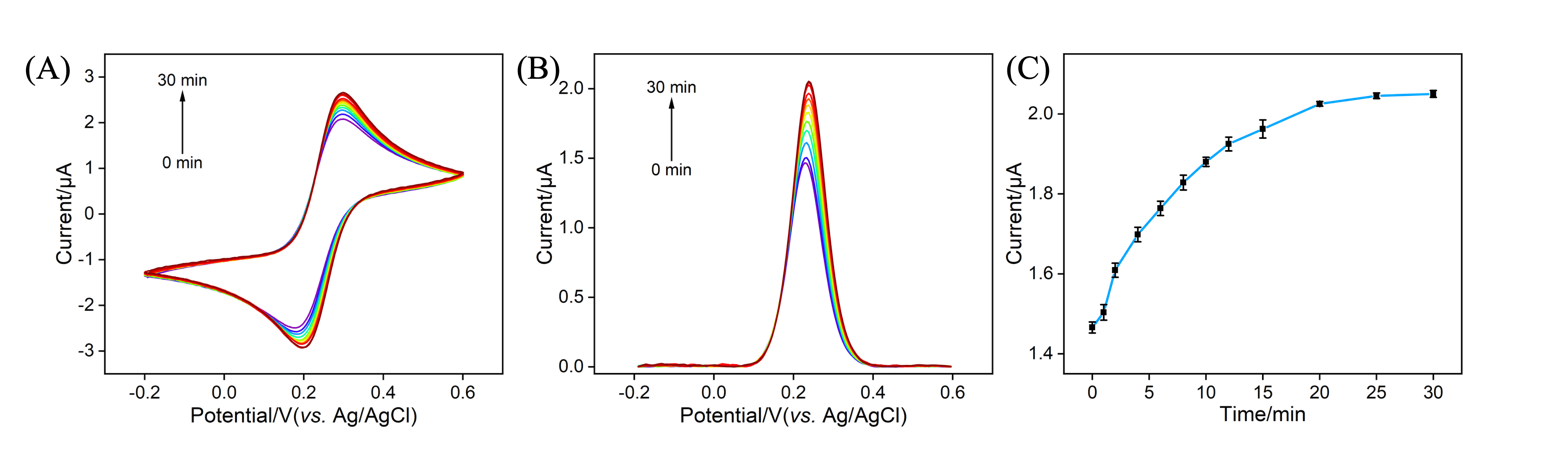
**

**Figure S1** CV(A) and DPV(B) of the VMSF/ITO electrode in 0.1 M KCl solution containing 0.05 mM Fe(CN)_6_^3−^ at different accumulation time with scan rate of 50 mV/s. (C) Peak current of the corresponding DPV curves at different accumulation times. Average values were calculated with three determinations.

**S2.** **Optimization of Fe(CN)_6_^3−^ concentration**

**
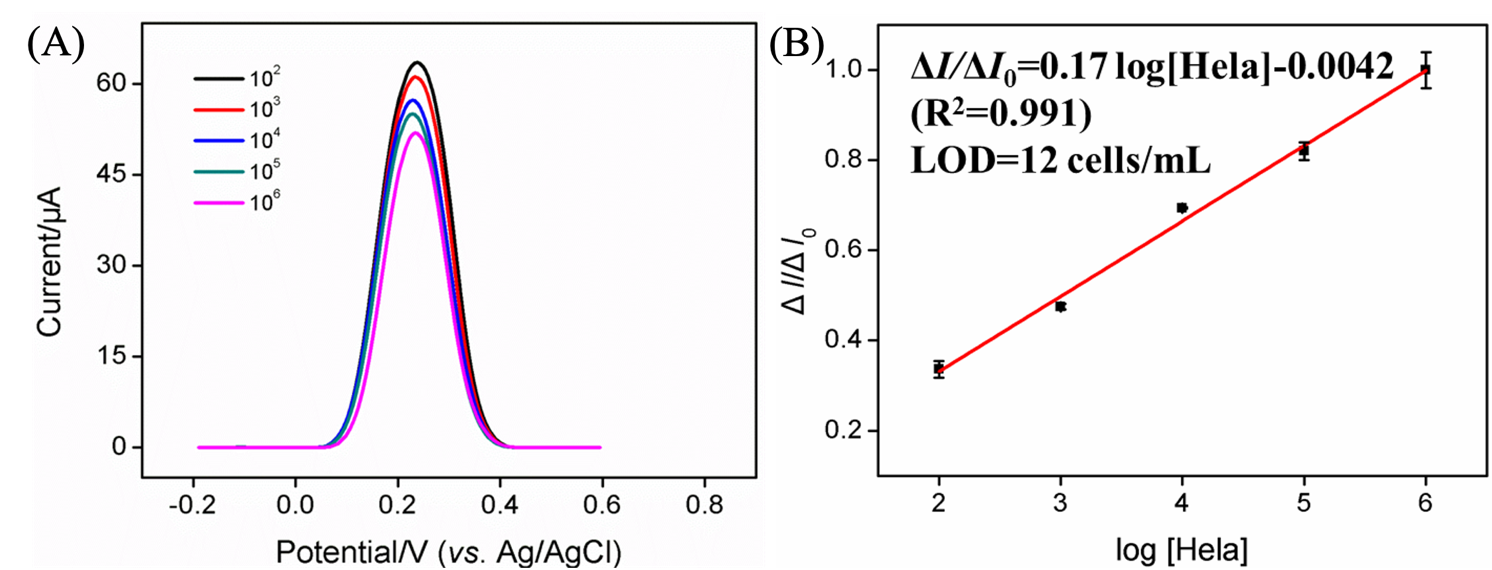
**

**Figure S2** (A) DPV curves of the FA-APTES/VMSF/ITO cytosensor in 0.5 mM K_3_[Fe(CN)_6_] and containing different concentrations of Hela cells. The concentration range is 10^2^−10^6^ cells/mL. (B) The corresponding calibration curves for the detection of Hela cells.

**S3. Selectivity of the FA-APTES/VMSF/ITO electrode**


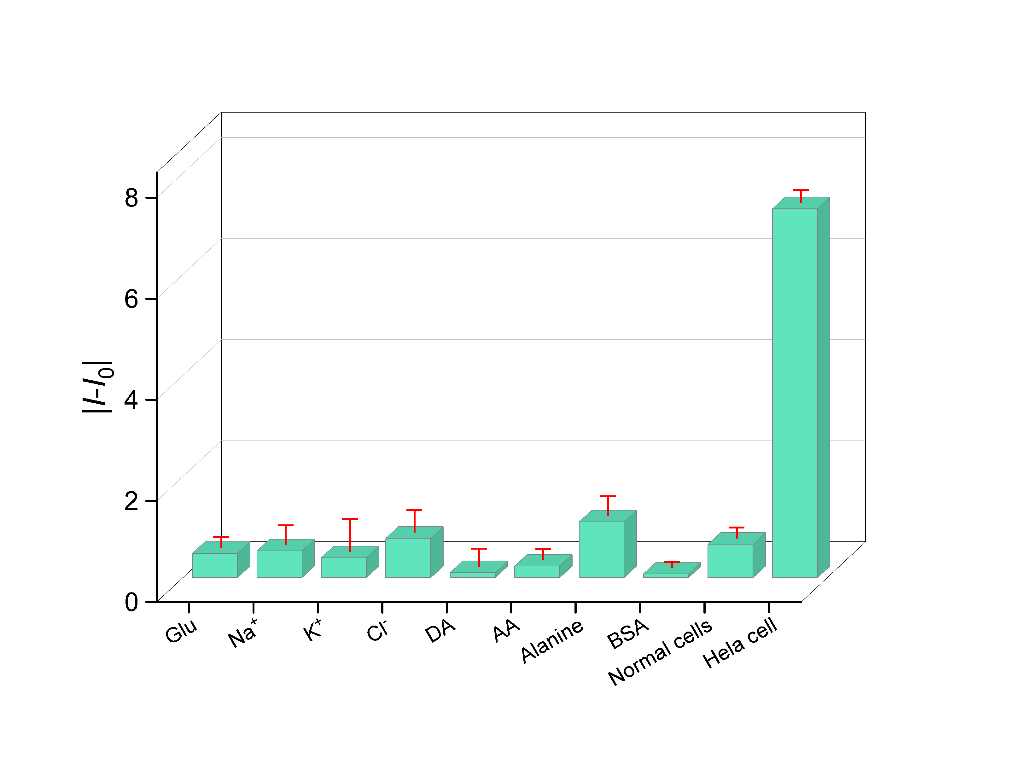


**Figure S3** The current response change value(|*I*−*I*_0_|) obtained from the FA-APTES/VMSF/ITO for detection of 10^4^ Hela cells in 0.1 M KCl solution containing 0.05 mM Fe(CN)_6_^3−^ in the absence (*I*_0_) and presence (*I*) of added interfering species. The concentration of Na^+^, K^+^, Cl^−^ and alanine is 10 mM, and the concentration of DA and AA is 50 μM. The concentration of BSA is 0.1%.
